# Society Meetings

**Published:** 1894-04

**Authors:** 


### Society Meetings.

THE Section on Surgery and Anatomy of the American Medical Association, of which Dr. John B. Roberts, of Philadelphia, is chairman, and Dr. F. W. McRae, of Atlanta, is secretary, will hold its meeting in San Francisco, June 5–8, 1894. It is proposed to devote a portion of the time of this section to the systematic consideration of a few selected subjects, upon which papers, each not occupying more than ten minutes, will be read. It is hoped that speakers discussing these papers will confine their remarks to brief addresses of five minutes' length. The topics and papers to be so presented are as follows: (1) malignant growths; (2) tubercular disease of joints; (3) hernia; (4) hemorrhoids, fistulæ and fissure; (5) fractures; (6) obstruction to urination in the male. Members who have specimens or patients to exhibit bearing on these topics, or who wish to make remarks in the discussion of them, are cordially invited to be present during the meetings of the section. The titles of other papers to be presented to the section will be published when the program of the meeting of the association is issued by the committee of arrangements.

---

THE Fourth Annual Meeting of the Association of Military Surgeons of the United States will be held in Washington, D. C., May 1, 2 and 3, 1894. This organization is composed of medical officers of the United States Army, Navy, Marine Hospital Service and the National Guard of the United States. Papers of interest to military surgeons will be read, the afternoon of one day will be set apart for a drill by the hospital corps, while the evenings will be devoted to social entertainments. Major George Henderson, M. D., Surgeon-General of the N. G. of D. C., 817 S. street, N. W. Washington, is the chairman of the committee of arrangements.

---

THE Forty-fifth Annual Session of the Medical Association of Georgia will meet in Atlanta, Ga., on April 18, 19 and 20, 1894. The officers are: President, W. H. Elliott, M. D., of Savannah; Vice-Presidents, G. T. Miller, M. D., of Americus, H. McHatton, M. D., of Macon; Treasurer, E. C. Goodrich, M. D., of Augusta; Secretary, Dan. H. Howell, M. D., of Atlanta.

---

THE Medical Society of the State of Pennsylvania will meet at Gettysburg, May 15, 1894. Dr. E. E. Montgomery of 1715 Walnut street, Philadelphia, is the chairman of the committee of arrangements.

---

THE Third Congress of American Physicians and Surgeons will be held in Washington, D. C., May 29th, 30th and 31st, and June 1, 1894, under the presidency of Dr. Alfred C. Loomis, of New York.

